# Gut Microbiota-Derived Metabolite and Heart Failure with Reduced Ejection Fraction (HFrEF): Elevated Trimethylamine N-Oxide (TMAO) as a Potential Biomarker

**DOI:** 10.3390/ijms27020703

**Published:** 2026-01-09

**Authors:** Sheh Wen Kuan, Wei Leik Ng, Alexander Loch, Kek Heng Chua, Kim-Kee Tan, Boon Pin Kee

**Affiliations:** 1 Department of Biomedical Science, Faculty of Medicine, Universiti Malaya, Kuala Lumpur 50603, Malaysia; kuansw0203@gmail.com (S.W.K.);; 2 Department of Primary Care Medicine, Faculty of Medicine, Universiti Malaya, Kuala Lumpur 50603, Malaysia; 3 Department of Medicine, Faculty of Medicine, Universiti Malaya, Kuala Lumpur 50603, Malaysia; 4 Tropical Infectious Diseases Research and Education Centre (TIDREC), Higher Institution Centre of Excellence (HICoE), Universiti Malaya, Kuala Lumpur 50603, Malaysia

**Keywords:** heart failure, TMAO, albumin, biomarker, ejection fraction, gut microbiota-derived metabolite

## Abstract

Gut-derived metabolites, particularly trimethylamine N-oxide (TMAO), have been implicated in the pathophysiology of heart failure (HF). This study investigated the associations between TMAO, cardiac function, and clinical parameters to evaluate TMAO’s potential as a biomarker for heart failure with reduced ejection fraction (HFrEF). Forty HFrEF patients and forty-one matched healthy controls were recruited for serum TMAO quantification using enzyme-linked immunosorbent assay (ELISA). Associations were examined using Spearman correlation and regression models. TMAO levels were significantly elevated in HFrEF patients (3.64 µM [IQR 3.00–4.31]) compared with controls (1.22 µM [IQR 0.92–2.36]) (*p* < 0.05). Elevated TMAO correlated with impaired cardiac structural and functional parameters, as well as lower serum albumin. Multinomial regression revealed that both TMAO (OR 1.83, 95% CI 1.04–3.23, *p* = 0.036; OR 2.05, 95% CI 1.18–3.57, *p* = 0.010, respectively) and albumin (OR 0.56, 95% CI 0.36–0.89, *p* = 0.015; OR 0.61, 95% CI 0.39–0.93, *p* = 0.022, respectively) were independently associated with HFrEF severity, showing significant correlations in both mildly (EF 30–40%) and moderately (20–30%) reduced EF groups. Receiver operating characteristic (ROC) analyses showed that TMAO had good discriminative ability for HFrEF (AUC = 0.853), and it improved when combined with clinical covariates (AUC = 0.967), supporting its role as a potential biomarker. These findings support integrating this gut-derived metabolite and nutritional marker into HFrEF risk stratification frameworks.

## 1. Introduction

Heart failure (HF) is defined as a structural or functional impairment of ventricular filling or ejection of blood, leading to elevated intracardiac pressures and/or inadequate cardiac output at rest and during exercise [1], and it is usually the end stage of cardiovascular diseases. This impairment leads to inadequate perfusion of peripheral tissues and vital organs. HF is commonly categorised based on left ventricular ejection fraction (LVEF) into distinct phenotypes: heart failure with reduced ejection fraction (HFrEF), defined by an LVEF ≤ 40%; heart failure with mildly reduced ejection fraction (HFmrEF), with LVEF between 41% and 49%; heart failure with preserved ejection fraction (HFpEF), where LVEF is ≥50% [2].

HF is a substantial burden on global health, with the Global Burden of Disease (GBD) Study 2023 estimating a prevalence of 56.02 million in 2023. The main causes of HF were ischemic heart disease, hypertensive heart disease, and cardiomyopathies. In Malaysia, 164,549 people were estimated to have HF in 2023, of whom 13,064 (7.94%) were caused by cardiomyopathies [3]. As populations age and the prevalence of risk factors such as hypertension and obesity increases, there is an urgent need for improved diagnostic and prognostic tools to enhance patient care.

Recent research has highlighted the role of the gut microbiota in cardiovascular health through the gut–heart axis [4,5]. In HF patients, splanchnic congestion driven by reduced cardiac output and elevated central venous pressure led to intestinal ischemia and increased gut permeability. This “leaky gut” phenomenon creates a self-perpetuating cycle of cardiac deterioration [6].

Crucially, dysbiosis could generate metabolites that may directly influence cardiac health. The major class of microbial metabolite, trimethylamine N-oxide (TMAO), has gained attention for its role in HF [7]. TMAO is produced by gut microbes (e.g., Firmicutes and Proteobacteria) when dietary precursors such as choline and L-carnitine are metabolised [8]. Increased plasma TMAO was correlated with higher HF-related mortality and hospitalisation, even after adjustment of traditional risk factors and cardiorenal indices [9,10]. TMAO accelerates HF progression by inducing cardiac hypertrophy and fibrosis [11], mitochondrial dysfunction [12], and inflammation [13]. This comprehensive involvement establishes TMAO as a central pathogenic mediator in the progression of heart failure.

To our knowledge, this is among the first studies to investigate the diagnostic potential of TMAO specifically in a Malaysian HFrEF cohort. This geographic validation is critical because TMAO production is contingent upon the interplay between dietary precursors and the gut microbiome. Prior studies have predominantly focused on Western populations with diets characterised by high red meat and dairy consumption. The Malaysian population, with its distinct multi-ethnic composition and dietary habits (rich in coconut milk, plant-based precursors, and spices), may harbour unique gut enterotypes that are different from Western cohorts [14]. Furthermore, while the link between TMAO and adverse outcomes is well-documented, its specific association with nutritional status (e.g., serum albumin) and clinical severity in a strictly defined HFrEF population requires further characterisation.

Therefore, this study aims to evaluate TMAO as a biomarker by comprehensively profiling serum TMAO and investigating its associations with cardiac function, nutritional markers, and disease severity in a Malaysian HFrEF cohort. To ensure robust and interpretable findings, the study employed an age- and gender-matched case–control design to control for demographic confounders, allowing for a more accurate attribution of metabolic differences to the HFrEF condition itself.

## 2. Results

### 2.1. Participant Characteristics

The baseline characteristics are shown in Table 1. A total of 40 HFrEF patients [34M, 6F; median age 60 (IQR 53.25–64) years] and 41 controls [33M, 8F; median age 63 (IQR 55–66) years] were recruited. The groups were well matched for age (Mann–Whitney U test, *p* = 0.334) and gender (Chi-square test, *p* = 0.808), as there were no significant differences. Among the vital signs, only heart rate differed significantly between groups (*p* = 0.024), while systolic and diastolic blood pressures were comparable (*p* = 0.977 and *p* = 0.265, respectively). For lifestyle and behavioural factors, there was no significant difference in overall smoking status (*p* = 0.108) (Supplementary Table S1), though patients reported a higher daily amount of cigarettes (*p* = 0.028). Alcohol consumption was significantly more common in controls (*p* = 0.002), with a higher weekly intake (*p* = 0.005). Physical activity of at least 30 min was significantly lower in patients (*p* < 0.001). Bowel habits and average sleep hours did not differ significantly between groups (*p* = 0.691 and *p* = 0.170, respectively). Their medical histories revealed that patients had a higher prevalence of prior coronary artery bypass grafting (CABG) (*p* = 0.004), percutaneous coronary intervention (PCI) (*p* < 0.001), myocardial infarction (*p* < 0.001), atrial fibrillation (*p* = 0.050), type 2 diabetes mellitus (T2DM) (*p* < 0.001) and chronic kidney disease (CKD) (*p* = 0.005). Dyslipidaemia was significantly more common in controls (*p* = 0.045). No significant differences were observed for stroke, hypertension, obesity, chronic obstructive pulmonary disease (COPD), anaemia, or gout. Patients had higher usage of angiotensin-converting enzyme inhibitors (ACEi) (*p* = 0.041), angiotensin receptor neprilysin inhibitor (ARNI) (*p* < 0.001), β-blockers (*p* < 0.001), mineralcorticoid receptor antagonist (MRA) (*p* < 0.001), diuretics (*p* < 0.001), sodium-glucose cotransporter 2 (SGLT2) inhibitors (*p* < 0.001), and antiplatelets (*p* < 0.001). Other medications, such as angiotensin receptor blockers (ARB), biguanides, calcium channel blockers, statins, and anticoagulants, showed no significant group differences.

### 2.2. TMAO Levels in HFrEF Patients and Control Group

**Figure 1:**
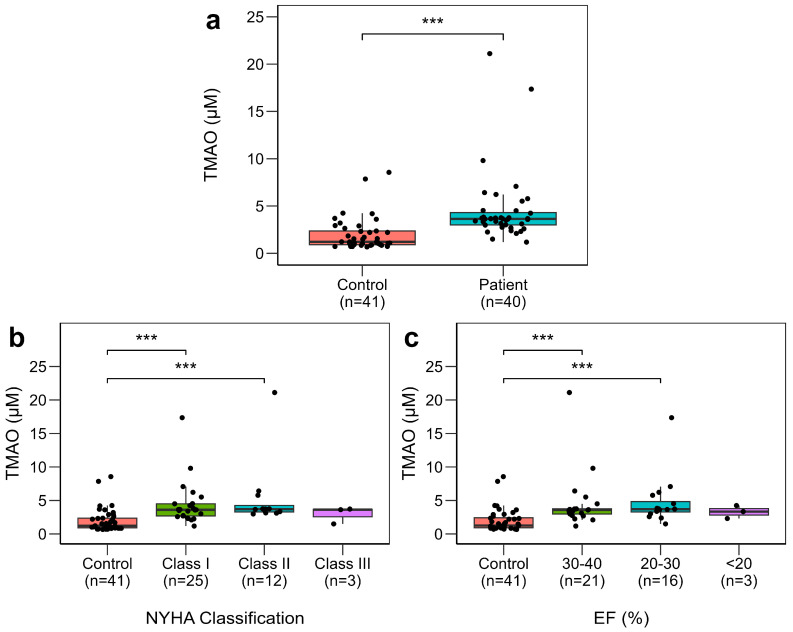
Box plots of serum metabolites in HFrEF patients and controls. (**a**) Serum TMAO. (**b**) Serum TMAO in HFrEF patients with NYHA functional classification (class I, II, and III) compared to controls. (**c**) Serum TMAO levels in HFrEF patients with different EF levels (30–40%, 20–30%, <20%) compared to controls (>40%). Box plots depict median (central line), interquartile range (IQR) (box), and range (whiskers). All data are visualised with median/IQR due to non-normality in Shapiro–Wilk test. *** indicates *p* < 0.001. TMAO; trimethylamine N-oxide, NYHA; New York Heart Association, EF; ejection fraction.

Serum TMAO levels were significantly elevated in HFrEF patients (median 3.64 μM, IQR 3.00–4.31) compared to controls (1.22 μM, IQR 0.92–2.36; *p* < 0.001) (Table 2; Figure 1a).

### 2.3. Associations of TMAO with Cardiac Structural and Functional Parameters and Albumin Levels

Spearman’s correlation analysis was performed to identify correlations between TMAO level and clinical factors (Supplementary Table S3). The results revealed that TMAO was strongly related to ejection performance and diastolic function. An elevated TMAO level was correlated with reduced EF measured by both the modified Simpson’s method (MOD-sp4) (r_s_ = −0.580) and the Teichholz (Teich) methods (r_s_ = −0.557), as well as reduced diastolic function (r_s_ = 0.416). In addition, TMAO levels also positively correlated with both end-diastolic volume (EDV) (MOD-sp4, r_s_ = 0.514; Teich, r_s_ = 0.464) and end-systolic volume (ESV) (MOD-sp4, r_s_ = 0.535; Teich, r_s_ = 0.555). Increased TMAO level also demonstrated a strong relationship with left ventricle (LV) chamber size, reflected by increased left ventricular end-diastolic diameter (LVEDD), left ventricular internal dimension at end-diastole (LVIDd), and left ventricular diameter at end-systole (LVIDs) (r_s_ = 0.489, 0.514, and 0.529, respectively). TMAO levels demonstrated a significant negative correlation with interventricular septum (IVS) fractional thickness (r_s_ = −0.450). A decrease in IVS fractional thickness indicates potential impairment in myocardial contractility. Notably, albumin was the only biochemical marker that showed a significant association with TMAO (r_s_ = −0.431), suggesting a potential link between reduced albumin concentrations and elevated TMAO. All associations reported met a significance threshold of *p* < 0.001 with |r_s_| > 0.4.

**Figure 2:**
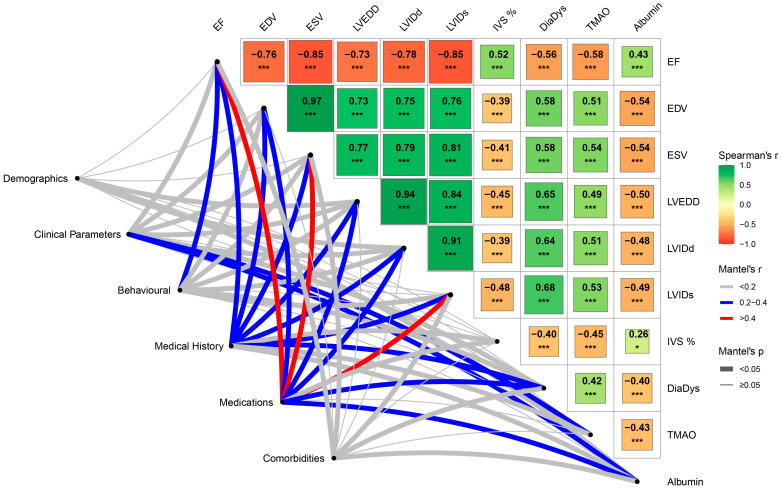
Spearman correlation and Mantel test between parameters significantly associated with TMAO in Spearman analysis (cardiac structure, function, and albumin) and grouped domains (demographics, clinical parameters, behavioural, medical history, medications, and comorbidities). The upper triangular heatmap displays the Spearman correlation coefficients between the TMAO and significant parameters, with colours indicating strength and direction (green for positive, red for negative) and square size reflecting correlation magnitude. Asterisks indicate statistical significance of the Spearman correlations (* *p* < 0.05, *** *p* < 0.001). Network lines overlay the matrix to highlight Mantel test results, where line thickness denotes *p*-value range (<0.05, ≥0.05) and colour reflects Mantel’s *r* (<0.2, 0.2–0.4, ≥0.4).

To further explore whether these associations were consistent with broader patient characteristics, Mantel tests were performed to compare the significant parameters from the Spearman analysis against grouped domains, including data regarding demographics, clinical parameters, behavioural factors, medical history, medications, and comorbidities. The Mantel analysis demonstrated several noteworthy patterns (Figure 2). Demographic factors showed only weak associations overall, though significant correlations were detected with LVEDD (r = 0.098, *p* = 0.042), LVIDd (r = 0.111, *p* = 0.021), and albumin (r = 0.191, *p* < 0.001). Clinical parameters revealed broader associations, with EF (r = 0.056, *p* = 0.035), EDV (r = 0.152, *p* = 0.036), ESV (r = 0.137, *p* = 0.038), LVEDD (r = 0.198, *p* = 0.019), LVIDd (r = 0.149, *p* = 0.030), diastolic dysfunction (r = 0.120, *p* = 0.012), and albumin (r = 0.401, *p* < 0.001) all significantly correlated, along with TMAO itself (r = 0.255, *p* = 0.035).

In the behavioural domain, significant correlations were observed with EF (r = 0.237, *p* < 0.001), EDV (r = 0.127, *p* = 0.004), ESV (r = 0.200, *p* < 0.001), LVEDD (r = 0.130, *p* = 0.002), LVIDd (r = 0.121, *p* = 0.002), LVIDs (r = 0.170, *p* < 0.001), and diastolic dysfunction (r = 0.069, *p* = 0.024), though no significant correlations with TMAO or albumin were identified in this group.

The medical history domain showed significant moderate correlations (*p* < 0.001), including EF (r = 0.361), EDV (r = 0.288), ESV (r = 0.371), LVEDD (r = 0.240), LVIDd (r = 0.363), LVIDs (r = 0.379), IVS fractional thickness (r = 0.113), and diastolic dysfunction (r = 0.278). Significant but weaker associations with TMAO (r = 0.156, *p* = 0.029) and albumin (r = 0.144, *p* = 0.020) were also observed.

The medications domain exhibited the moderate associations overall (*p* < 0.001), with EF (r = 0.503), EDV (r = 0.325), ESV (r = 0.459), LVEDD (r = 0.221), LVIDd (r = 0.314), LVIDs (r = 0.447), and diastolic dysfunction (r = 0.284). Albumin was also correlated with this domain (r = 0.219, *p* < 0.001).

Comorbidities showed more modest associations, with significant correlations observed for EF (r = 0.069, *p* = 0.006), ESV (r = 0.099, *p* = 0.040), LVIDs (r = 0.062, *p* = 0.046), and diastolic dysfunction (r = 0.077, *p* = 0.045). Albumin was borderline significant (r = 0.110, *p* = 0.049), while TMAO was not significantly correlated.

### 2.4. Logistic Regression Analysis of Predictors for HFrEF

Univariate logistic regression was used to assess the individual predictive capability of each parameter in identifying HFrEF. A total of 30 variables were found to show a robust association with HFrEF (*p* < 0.05). These included patient characteristics (ethnicity, heart rate), lifestyle factors (alcohol consumption), clinical diagnoses (dyslipidaemia, T2DM), a wide array of biochemical markers (including HbA1c, renal function tests, and lipid profiles), and extensive echocardiographic parameters related to cardiac structure and function such as ejection fraction, ventricular volumes, and wall thickness (Table 3). Of note, TMAO (OR 2.13, 95% CI:1.48–3.31, *p* < 0.001) showed a strong association with HFrEF.

To isolate the most critical variables independently associated with HFrEF, multivariate logistic regression was used. After adjusting for covariates in the final model, three variables retained their statistical significance. The strong association of TMAO with the outcome was particularly noteworthy. Specifically, each 1 µM elevation in TMAO was associated with an 85% higher likelihood of HFrEF (OR 1.85, 95% CI: 1.20–3.37, *p* = 0.016). In contrast, higher serum albumin was inversely associated with HFrEF, with each g/L increase corresponding to a 42% reduction in the odds of having HFrEF (OR 0.58, 95% CI: 0.36–0.83, *p* = 0.008), while the presence of diastolic dysfunction was strongly associated with HFrEF (OR 5.19, 95% CI: 1.84–22.50, *p* = 0.007).

### 2.5. Evaluation of Discriminative Ability of TMAO as a Candidate HFrEF Biomarker Using ROC Analysis

**Figure 3:**
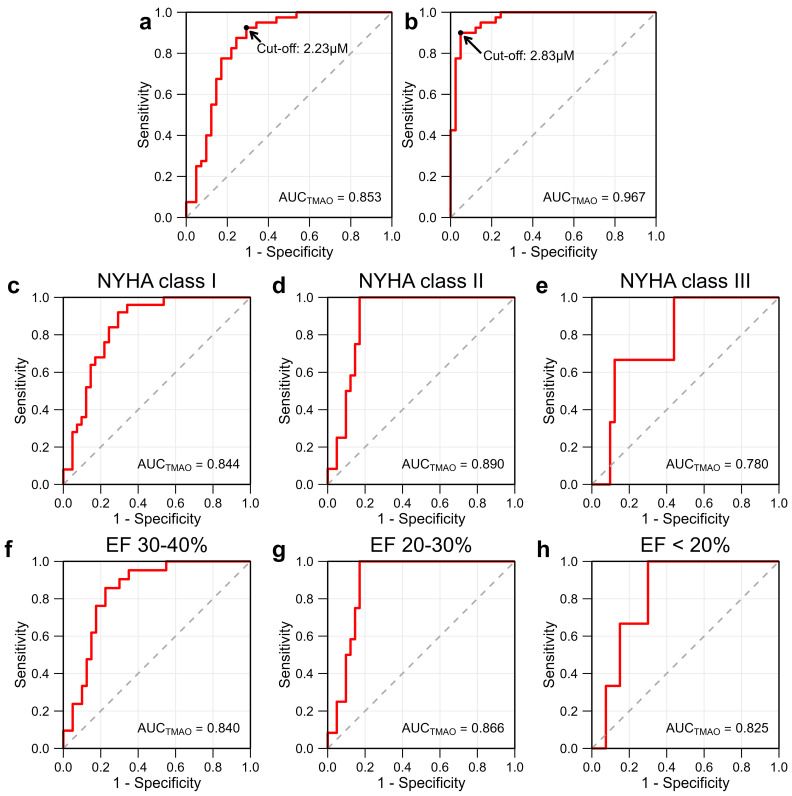
Receiver operating characteristic (ROC) analyses of TMAO in relation to HFrEF and HF severity. (**a**) ROC curve of TMAO for prediction of HF. (**b**) ROC curve of TMAO with adjustment for multiple covariates. (**c**–**e**) ROC curves of TMAO according to NYHA functional classification: (**c**) NYHA class I, (**d**) NYHA class II, and (**e**) NYHA class III. (**f**–**h**) ROC curves of TMAO according to EF levels: (**f**) mildly reduced (30–40%), (**g**) moderately reduced (20–30%), (**h**) severely reduced (<20%). The area under the curve (AUC) is indicated in each panel.

ROC analysis was performed to evaluate the ability of TMAO to distinguish HFrEF patients from controls. The area under the curve (AUC) for TMAO was 0.853 (95% CI: 0.765–0.941) (Figure 3a), indicating that TMAO had a good ability to discriminate HFrEF patients from controls. At the cutoff threshold of 2.23 µM, TMAO could yield a maximum sensitivity of 92.5% and specificity of 70.7%. To determine whether the association of TMAO was independent of other clinical factors, covariates were incorporated for ROC analysis. The AUC increased to 0.967 (95% CI 0.933–1.000), with a sensitivity of 90.0% and specificity of 95.1% at the threshold of 2.83 µM (Figure 3b), indicating improved discriminative performance. The results suggest that TMAO exhibited strong potential as a candidate biomarker for HFrEF.

### 2.6. TMAO Levels and HFrEF Severity

To further investigate whether TMAO levels varied with disease severity, TMAO levels were compared across controls and New York Heart Association (NYHA) functional classification. The Kruskal–Wallis test revealed an overall significant difference among groups (*p* < 0.001). Post-hoc pairwise comparisons showed that TMAO levels were significantly higher in the NYHA class I (*p* < 0.001) and class II (*p* < 0.001) compared to controls (Figure 1b; Supplementary Table S4). Notably, the associations remained significant (*p* < 0.001) even after adjustment by Bonferroni correction. In contrast, the difference for the NYHA class III did not reach statistical significance, likely due to the smaller sample size in this subgroup.

ROC analysis was employed to further determine the discriminative performance of TMAO across NYHA functional classification. TMAO demonstrated a good discriminative ability of NYHA class I and class II, both with AUC values > 0.8 (0.844, 95% CI 0.765–0.941 and 0.890, 95% CI: 0.803–0.977, respectively) (Figure 3c–e). For NYHA class I, at a threshold of 2.37 µM, TMAO could yield a maximum sensitivity of 92.0% and specificity of 70.7%. For NYHA class II, at a threshold of 2.96 µM, TMAO could yield a maximum sensitivity of 100.0% and specificity of 82.9%. Meanwhile, for NYHA class III, the AUC was slightly lower (0.780, 95% CI: 0.545–1.000), with a sensitivity of 100% and specificity of 56.1% at a threshold of 3.61 µM. These findings suggest that TMAO had strong discriminative value in early NYHA classes (I and II), whereas the lower AUC observed in NYHA class III could be due to the smaller number of patients in this subgroup.

To complement these findings, the association of TMAO with LV systolic function was carried out by categorising patients according to EF levels: mildly reduced EF (30–40%), moderately reduced EF (20–30%), and severely reduced EF (<20%). Patients with moderately reduced EF (20–30%) and mildly reduced EF (30–40%) had significantly higher TMAO compared with controls, and this significance remained (*p* < 0.001) even after adjustment by Bonferroni correction (Figure 1c). However, there was no significant difference between severely reduced EF (<20%) and TMAO level.

In addition, ROC analysis further supported the discriminatory performance of TMAO across EF levels. TMAO demonstrated a good discriminative ability of all EF levels, all with AUC values > 0.8 (0.840, 95% CI 0.741–0.940; 0.866, 95% CI: 0.770–0.961 and 0.825, 95% CI 0.663–0.987, respectively) (Figure 3f–h). For mildly reduced EF (30–40%), at the threshold of 2.67 µM, TMAO could yield the maximum sensitivity of 85.7% and specificity of 77.5%. For moderately reduced EF (20–30%), at the threshold of 2.97 µM, TMAO could yield the maximum sensitivity of 93.8% and specificity of 75.0%. For severely reduced EF (<20%), at the threshold of 3.44 µM, TMAO could yield the maximum sensitivity of 100.0% and specificity of 70.0%. These findings suggest that TMAO had strong discriminative value in all EF levels.

To account for potential confounding factors, multinomial logistic regression was conducted. In the analysis, all individuals with preserved EF (>40%) belonged to the control group, which served as the reference category. The results showed that higher TMAO levels were independently associated with both mildly reduced EF (30–40%) and moderately reduced EF (20–30%) (Table 4). Specifically, for every 1 µM increase in TMAO, the odds of having EF 30–40% increased 83% (OR 1.83, 95% CI: 1.04–3.23, *p* = 0.036), while the odds of having EF 20–30% increased more than two-fold (OR 2.05, 95% CI 1.18–3.57, *p* = 0.010), compared with controls (EF > 40%). Meanwhile, TMAO showed no significant association with severely reduced EF (<20%) (OR 0.84, 95% CI 0.21–3.35, *p* = 0.807).

In addition to TMAO, lower albumin levels were also significantly associated with mildly reduced EF (30–40%) (OR 0.56, 95% CI 0.36–0.89, *p* = 0.015), suggesting a possible link between inflammatory status and systolic dysfunction. This association was also observed in the moderately reduced EF (20–30%) group (OR 0.61, 95% CI 0.39–0.93, *p* = 0.022) but did not persist in the severely reduced EF (<20%) group (OR 0.61, 95% CI 0.28–1.33, *p* = 0.212).

Diastolic dysfunction showed a strong association with mildly and moderately reduced EF. Patients with mildly reduced EF (30–40%) were associated with six times greater odds of diastolic dysfunction (OR 5.83, 95% CI 1.56–21.82, *p* = 0.009), and those with moderately reduced EF (20–30%) demonstrated an association with 5-fold higher odds (OR 5.38, 95% CI 1.46–19.85, *p* = 0.011). However, this association was not significant in the severely reduced EF (<20%) group (OR 5.27, 95% CI 0.59–46.85, *p* = 0.136). The magnitude of diastolic dysfunction effect estimates may partly reflect collinearity with EF and small subgroup sizes, warranting cautious interpretation. Other covariates, including age, gender, alcohol intake, estimated glomerular filtration rate (eGFR), haemoglobin A1c (HbA1c), and high-density lipoprotein (HDL), did not demonstrate significant associations.

## 3. Discussion

This study revealed a consistent and reproducible relationship between TMAO and HFrEF. TMAO was significantly associated with HFrEF, as the TMAO level was highly elevated in HFrEF patients as compared to the controls, consistent with previous studies [15,16,17]. Higher TMAO levels were associated with adverse echocardiographic indices, worse NYHA functional class, and lower EF. Even after adjustment for covariates such as age, gender, eGFR, alcohol intake, HbA1c, HDL, diastolic dysfunction, and albumin, strong significance remained between TMAO and HFrEF. This highlighted TMAO as a marker that is independently associated with HFrEF and its severity. Results showed that elevated TMAO was associated not only with the presence of HFrEF but also with worsening ventricular function, particularly in the earlier to moderate stages of disease progression. In the subgroup analysis across different EF levels, TMAO consistently emerged as a significant predictor for patients with mildly reduced EF (30–40%) and moderately reduced EF (20–30%), with odds ratios of 1.83 and 2.05, respectively. However, the TMAO levels did not correlate linearly with the NYHA class III and severely reduced EF (<20%) group, which was in contrast with reported studies [18,19]. This may be partly attributable to the small number of patients in this subgroup, which reduces statistical power and increases susceptibility to type II error. Additionally, patients with extremely low EF often have a higher comorbidity burden, renal impairment, or prior HF events that may affect circulating TMAO, introducing survivor bias. Extreme HF may trigger compensatory metabolic changes, altered gut microbiota, or cachexia that obscure simple correlations. In addition, TMAO also demonstrated strong discriminative performance in ROC analysis. Specifically, higher TMAO levels distinguished patients with HFrEF from controls with a high AUC score, highlighting its potential utility as a candidate biomarker.

These findings aligned with a study reporting that TMAO could be used to predict cardiovascular death in HFrEF patients [16]. In addition, TMAO was identified as a strong prognostic marker in chronic HF, showing consistent associations with diastolic dysfunction and adverse long-term outcomes [19]. In the BIOlogy Study to TAilored Treatment in Chronic Heart Failure (BIOSTAT-CHF) study, HF patients with higher TMAO levels had higher mortality and hospitalisation rates [20]. Microbial genes for choline TMA-lyase, which are involved in TMAO production, were upregulated in HF patients, further supporting the link between gut-derived TMAO production and HF [15].

Elevated TMAO contributed to progressive ventricular remodelling (cardiac hypertrophy and fibrosis) and HF in many plausible ways. TMAO could promote chronic inflammation through the activation of nuclear factor kappa B (NF−κB) and NOD-like receptor protein 3 (NLRP3) inflammasomes, which impair endothelial function and promote vascular injury, ultimately exacerbating HF [21]. In addition, TMAO could impair myocardial energetics by disruption of pyruvate and fatty acid oxidation, thereby contributing to ventricular remodelling and progression of HF [12]. Animal studies showed that a diet high in TMAO worsened HF by inducing left ventricular dilation and myocardial fibrosis [11,22]. The involvement in inflammatory and metabolic pathways provided a strong biological plausibility for the significant association that had been observed between TMAO levels and the severity of HFrEF in this study.

Dietary intake is an important determinant of circulating TMAO, and this factor warrants careful consideration in the interpretation of the current findings. Numerous studies have demonstrated that consumption of fish, red meat, and eggs leads to a substantial postprandial increase in circulating TMAO levels due to enhanced substrate availability for gut microbial TMA production and subsequent hepatic oxidation. However, several pieces of evidence suggest that diet alone did not fully account for the TMAO variation observed in HF. Prior HF and coronary artery disease cohorts have similarly demonstrated significant associations between elevated TMAO and clinical outcomes despite the absence of detailed dietary records [9,16,23]. This suggests that non-dietary factors, including gut microbiota composition, hepatic FMO3 activity, renal clearance, systemic inflammation, and HF-related metabolic alterations, contributed substantially to circulating TMAO levels. Consistent with this, we observed that TMAO correlated with cardiac structural and functional impairment and was associated with HF severity categories in multivariable models that included clinical covariates. These findings indicate that while dietary variation may introduce measurement variability, it is unlikely to be the principal driver of the consistent and robust associations observed in this cohort.

Given the high utilisation of guideline-directed medical therapies (GDMT) such as ARNI, β-blockers, and SGLT2 inhibitors in the current HFrEF cohort, it is crucial to consider their potential impact on circulating TMAO levels. While these agents exert profound haemodynamic and metabolic effects, previous evidence suggested that TMAO was relatively resistant to standard pharmacological modulation. The BIOSTAT-CHF study demonstrated that guideline-based drug therapy did not affect TMAO levels [20]. Consequently, the prognostic value of TMAO likely persisted regardless of the patient’s medication regimen, supporting its utility as a complementary biomarker in HFrEF populations. Nevertheless, medication-related modulation of TMAO cannot be fully excluded and should be further explored in longitudinal and mechanistic studies. 

Remarkably, albumin was independently linked to HFrEF in this study and retained significance in the adjusted model (*p* < 0.008). In addition, albumin was also significantly associated with mildly reduced EF (30–40%) and moderately reduced EF (20–30%) groups. It was plausible that in the earlier phases, albumin decline could still differentiate patients from controls, as the variation in nutritional and inflammatory status across individuals was sufficient to identify patients at risk of progression. However, as the disease progressed and EF dropped below 20%, a lower level of albumin (hypoalbuminaemia) became common among patients due to the combined effects of chronic inflammation, fluid overload, and malnutrition, reducing its discriminative power between strata. This observation indicated that hypoalbuminaemia was common in HFrEF and may function more as a general marker of disease burden than as an EF level predictor at later stages. Meta-analysis reported hypoalbuminaemia as a HF prognostic marker, as it was consistently correlated with HF adverse prognoses, such as rehospitalisations and a high mortality rate [24]. Hypoalbuminaemia was more prominent in patients with NYHA class IV (69%) than those with lower classes (21%). Patients with hypoalbuminaemia were 1.8 times more likely to die, with a one-year survival rate of only 66% compared to 83% in those with a normal albumin level [25]. The prevalence of hypoalbuminaemia in HF was estimated to be 12% to 54% [26], with a particularly high prevalence of 69.6% observed in HFrEF [27].

The present study revealed that the co-occurrence of both albumin and TMAO as independent indicators associated with HFrEF was particularly noteworthy, as the interplay between these two markers had remained unexplored in HF research. While our cross-sectional study did not demonstrate a direct interaction between these biomarkers, their concurrent significance raises the possibility that they may reflect related or convergent pathophysiological processes. Several experimental and biophysical studies provided a foundational basis for this proposed link. Albumin was reported to have the capacity to bind small circulating molecules, including TMAO. This interaction was further supported by spectroscopic quenching and computational docking studies, which demonstrated a direct TMAO–albumin interaction [28]. Such findings have led to the hypothesis that albumin may act as a carrier or buffering molecule for TMAO. When TMAO was bound to albumin, it was less available to exert its direct cardiotoxic and pro-atherogenic effects. In the context of HF, with less albumin available, the concentration of free-circulating TMAO increased. The unbound TMAO could intensify its negative impacts on the cardiovascular system and contribute to HF progression.

In addition, it was reported that TMAO may influence the handling of albumin at the renal level. The kidneys could reabsorb filtered albumin to prevent its excretion in urine. This process was largely mediated by a key protein known as megalin, which was expressed on the surface of proximal tubular cells in the kidney. TMAO has been shown to downregulate megalin expression, thereby reducing albumin uptake in proximal tubular cells in vitro [29]. This led to an increase in albumin excretion in the urine. In short, TMAO could cause the kidneys to “leak” albumin. This mechanism could plausibly create a detrimental feedback loop: elevated TMAO may lead to albumin loss, and the subsequent hypoalbuminaemia could reduce the body’s ability to buffer the already-high TMAO. However, this concept remains theoretical and has not been validated in human HF cohorts. Further mechanistic studies and longitudinal clinical investigations are required to clarify the biological relevance of these biomarkers within the pathophysiology of HF.

A notable and interesting finding was observed in this study, whereby the cholesterol levels (total cholesterol, triglycerides, LDL, HDL, and non-HDL) of HFrEF patients were significantly lower than those of controls. Notably, statin usage was prevalent in both the HFrEF and control groups, suggesting that pharmacotherapy alone does not fully account for the magnitude of lipid reduction observed in HF patients. Conventionally, elevated cholesterol was recognised as the major cardiovascular risk factor. However, in the setting of HF, the opposite phenomenon was observed: lower cholesterol levels were associated with worse prognosis and increased mortality, giving rise to the term “cholesterol paradox” [30]. Systemic inflammation and metabolic disturbance were common in chronic HF, which led to the downregulation of hepatic cholesterol synthesis and increased lipoprotein catabolism [31]. In addition, HF patients were prone to develop cachexia and malnutrition, leading to a failure to support metabolic function, including the synthesis of lipids. In view of reduced cardiac performance in HF, the body enters a hypermetabolic state to compensate for the failing heart by breaking down its own fat and muscle stores for energy [32]. This causes a reduction in overall lipid mass and a corresponding drop in circulating cholesterol. Importantly, low cholesterol was not protective against HF but instead a surrogate marker for the severity of HF progression.

This study has several limitations. First, dietary intake of TMAO precursors was not assessed or controlled. Serum TMAO levels are influenced not only by disease status but also by dietary intake, especially red meat, fish, and fibre. Without detailed dietary records, the possibility that inter-individual dietary differences confounded the observed associations cannot be fully excluded. Future studies should incorporate validated dietary assessment tools (e.g., food-frequency questionnaires, 24 h dietary recalls) to better control for diet-related influences. Secondly, although multivariate models were adjusted for key clinical covariates, including eGFR, residual confounding cannot be completely excluded. Renal function is a major determinant of circulating TMAO concentrations. However, the influence of advanced renal impairment in the present cohort was limited, as only two patients in the HFrEF group had advanced CKD (eGFR < 30 mL/min/1.73 m^2^), suggesting severe renal dysfunction is unlikely to be the primary driver of the observed associations. Thirdly, although the overall cohort was moderate, the number of patients in certain subgroups (e.g., NYHA class III and EF < 20%) was relatively small. These patients are often more fragile and prone to complications, which made recruitment challenging. This limited sample size reduced the statistical power of subgroup analyses, potentially leading to underestimating or overestimating the discriminative performance of TMAO in more severe HFrEF categories. Future investigations should employ larger, multi-centre cohorts for broader validation. Finally, serum TMAO was quantified using a sandwich ELISA assay rather than the gold-standard LC-MS/MS. While LC-MS/MS offers superior specificity and sensitivity, ELISA was selected for its cost-effectiveness, high throughput, and accessibility in standard clinical laboratories, aligning with our aim to evaluate TMAO’s potential as a biomarker. Future studies would benefit from cross-validation using LC-MS/MS to confirm and extend the findings.

## 4. Materials and Methods

### 4.1. Study Population

A total of 81 individuals from University Malaya Medical Centre (UMMC), Kuala Lumpur, Malaysia, were recruited between January 2024 and March 2025. This study enrolled 40 patients with heart failure with reduced ejection fraction (HFrEF) and underlying cardiomyopathy diagnosed by cardiologists based on the clinical and echocardiographic guidelines from the American College of Cardiology/American Heart Association (ACC/AHA). All the HF patients had reduced left ventricular ejection fraction (LVEF) of ≤40% confirmed by transthoracic echocardiography. They were further classified into functional class I to III according to the New York Heart Association (NYHA). HF patients with hypertrophic cardiomyopathy were excluded from the study. Forty-one individuals with normal echocardiography and no clinical symptoms of cardiovascular diseases were included as the control cohort. Controls were selected to be comparable to cases with respect to age and gender. The exclusion criteria for both HFrEF and control cohorts were: age below 18 and above 70 years, use of antibiotics in oral, intravenous, or topical form within the past three months, history of inflammatory bowel diseases, irritable bowel syndrome, chronic diarrhoea, or other gastrointestinal disorders, use of immunosuppressive medications or chemotherapy, adherence to specific diet such as vegan, vegetarian, gluten-free and milk-free diet. The study was approved by the Medical Research Ethics Committee UMMC (MRECID no.: 202352-12411) and complied with the Declaration of Helsinki. Written informed consent was obtained from all individuals.

### 4.2. Clinical Data Collection and Lifestyle Questionnaire

Clinical data such as demographics, medical and medication history, laboratory results, and echocardiographic measurements were collected. Upon enrolment, all participants completed a structured, interviewer-administered questionnaire to capture key lifestyle factors known to influence cardiovascular and metabolic health. Smoking status, alcohol consumption, weekly exercise frequency, bowel-habit regularity, and sleep duration of each individual were documented. Questionnaire responses were coded, and categorical variables (e.g., smoking status) and continuous variables (e.g., frequency/week) were subsequently included in downstream statistical models to adjust for lifestyle factors.

### 4.3. Sample Collection

Venous blood was collected in a red plain tube and centrifuged at 3500 rpm for 10 min to obtain serum. Serum was aliquoted and stored at −80 °C until analysis.

### 4.4. Quantification of Serum TMAO Levels

The serum TMAO level was quantified using the Human TMAO enzyme-linked immunosorbent assay (ELISA) Kit from ELK Biotechnology [Wuhan, China] (ELK8356). All assays were performed according to the manufacturer’s instructions. All samples and standards were assayed in duplicate to ensure precision.

### 4.5. Statistical Analysis

All statistical analyses were performed using R version 4.4.2. The normality of data distribution was evaluated using the Shapiro–Wilk test. A two-sided *p* < 0.05 was considered statistically significant unless otherwise specified. Continuous variables were shown as mean ± SD and analysed with an independent Student’s *t*-test for normal distribution variables, or medians with interquartile ranges (IQR) and analysed with Mann–Whitney U test for non-normal distribution variables. Categorical variables were presented as frequencies (%) and were analysed using Chi-Square or Fisher’s exact tests as appropriate. The Receiver Operating Characteristic (ROC) curve was used to assess the discriminative ability of TMAO in HFrEF. The Kruskal–Wallis test followed by post hoc pairwise comparisons with correction was used to evaluate the association between TMAO and HFrEF severity.

Spearman’s rank test was performed for correlation analysis, as the data exhibited non-normal distributions (Shapiro–Wilk *p* < 0.05). To minimise false positives, a strict threshold of significance was defined as *p* < 0.001 and |r_s_| > 0.4. Significant variables from this analysis were further examined using the Mantel test to explore relationships across higher-level domains such as demographics, clinical parameters, behavioural, medical history, medications, and comorbidities. A full list of parameters included in each domain is provided in Supplementary Table S5.

For risk factor evaluation, logistic regression analysis was applied. In the univariate models, variables with complete separation were analysed using Firth’s penalised regression. For the multivariate model, purposeful selection was applied to prevent overfitting, whereby variables with *p* < 0.1 in univariate analysis were initially considered, followed by assessment of collinearity to remove variables with correlation coefficients > 0.7. Next, variables were reintroduced if their removal resulted in a >20% change in the coefficient (∆β) of remaining predictors, indicating an important confounding effect [33]. The final multivariate model included age, gender, eGFR, alcohol consumption, HbA1c, HDL, diastolic dysfunction, albumin, and TMAO to evaluate variables independently associated with HFrEF. Non-convergence occurred in specific instances due to extreme data sparsity and complete separation. As this precludes the mathematical estimation of relative risk against the baseline, valid Odds Ratios could not be generated for these specific strata and are reported as “Not converged”.

Multinomial regression was constructed to evaluate the associations between TMAO and HFrEF severity. The same final set of covariates identified in the multivariate analysis was included to adjust for potential confounding. The EF levels were defined as mildly reduced EF (30–40%), moderately reduced EF (20–30%), severely reduced EF (<20%), and preserved EF (>40%, controls).

## 5. Conclusions

In summary, the robust association between elevated TMAO and HFrEF suggests that TMAO is closely related to disease presence and severity. These findings highlight the potential of TMAO as a candidate biomarker to complement diagnosis and risk stratification in HFrEF, warranting further investigation into its utility for longitudinal monitoring. While TMAO remained significant in multivariable models adjusted for key clinical covariates, these findings should be interpreted in the context of the study’s limitations, including the modest sample size, small numbers in certain severity subgroups, the cross-sectional design, and the potential for residual confounding. As such, the observed relationships indicate association rather than causation. Furthermore, a significant association between albumin and HFrEF, a traditional marker of disease severity, was observed in this study. The significant findings for both albumin and TMAO suggest a novel and complex interplay between these two seemingly distinct biomarkers. While not directly correlated in our study, both markers may reflect convergent pathogenic pathways in HFrEF pathophysiology, warranting further investigation into their potential synergistic roles. These results contribute to a deeper understanding of the biological underpinnings of HFrEF and suggest that a multi-marker approach, incorporating both traditional and novel gut-derived metabolites, may provide a more comprehensive framework for disease characterisation.

## Figures and Tables

**Table 1:** Demographics, lifestyle and behaviour, medical history, and medications of the patient and control groups.

Characteristics	HFrEF (N = 40)	Control (N = 41)	*p* Value
**Demographics**			
Age, years	60.00 (53.25–64.00)	63.00 (55.00–66.00)	0.334
Male, n (%)	34 (82.93)	33 (80.49)	0.808
BMI, kg/m^2^	26.17 (23.54–30.59)	24.49 (23.14–29.05)	0.255
Ethnicity, n (%)			0.070
Malay	22 (55.00)	12 (29.27)	
Chinese	12 (30.00)	20 (48.78)	
Indian	5 (12.50)	9 (21.95)	
Eurasian	1 (2.50)	0 (0.00)	
NYHA class I/II/III, n (%)	25/12/3 (62.50/30.00/7.50)	N/A	N/A
Systolic BP, mmHg	129 (117–145)	130 (122–138)	0.977
Diastolic BP, mmHg	75 ± 12	78 ± 11	0.265
Heartrate, bpm	75.50 (68.75–83.50)	70.00 (64.00–78.00)	0.024 *
**Lifestyle and Behaviour, n (%)**			
Number of cigarettes, sticks/day	3.50 (0.00–15.50)	0.00 (0.00–5.00)	0.028 *
Alcohol			0.002 *
Former	4 (10.00)	2 (4.88)	
Current	5 (12.50)	20 (48.78)	
Never	31 (77.50)	19 (46.34)	
Frequency of alcohol consumption/week			0.005 *
Rare (<1)	5 (55.56)	20 (90.91)	
Occasional (1–2)	3 (33.33)	1 (4.55)	
Regular (3–6)	0 (0.00)	1 (4.55)	
Daily (7)	1 (11.11)	0 (0.00)	
Physical activity for ≥30 min			<0.001 *
<1 session/week	29 (72.50)	19 (46.34)	
1–3 sessions/week	8 (20.00)	12 (29.27)	
≥4 sessions/week	3 (7.50)	10 (24.39)	
**Medical history, n (%)**			
History of CABG	9 (22.50)	0 (0.00)	0.004 *
History of PCI	17 (42.50)	0 (0.00)	<0.001 *
History of myocardial infarction	25 (62.50)	0 (0.00)	<0.001 *
Atrial fibrillation	3 (7.50)	0 (0.00)	0.050 *
Dyslipidaemia	28 (70.00)	37 (90.24)	0.045 *
T2DM	28 (70.00)	10 (24.39)	<0.001 *
CKD	27 (67.50)	18 (43.90)	0.005 *
**Medications, n (%)**			
ACEi	16 (40.00)	7 (17.07)	0.041 *
ARNI	22 (55.00)	0 (0.00)	<0.001 *
β-blocker	35 (87.50)	6 (14.63)	<0.001 *
MRA	31 (77.50)	0 (0.00)	<0.001 *
Diuretics	26 (65.00)	1 (2.44)	<0.001 *
SGLT2 inhibitor	36 (90.00)	1 (2.44)	<0.001 *
Antiplatelet	24 (60.00)	5 (12.20)	<0.001 *

Continuous variables are presented as mean ± SD or medians with interquartile ranges (IQR), depending on the distribution of the data. ACEi, angiotensin-converting enzyme inhibitor; ARNI, angiotensin receptor-neprilysin inhibitor; BMI, body mass index; BP, blood pressure; CABG, coronary artery bypass grafting; CKD, chronic kidney disease; HFrEF, heart failure with reduced ejection fraction; MRA, mineralocorticoid receptor antagonist; NYHA, New York Heart Association; PCI, percutaneous coronary intervention; SGLT2 inhibitor, sodium-glucose cotransporter 2 inhibitor; T2DM, Type 2 diabetes mellitus. * indicates *p* < 0.05. Note: Demographics are listed regardless of significance. For other parameters, only variables with statistically significant differences (*p* < 0.05) are displayed. The complete list of all analysed parameters, including non-significant associations, is available in Supplementary Table S1.

**Table 2:** Laboratory and echocardiographic parameters.

Parameters	HFrEF (N = 40)	Control (N = 41)	*p* Value
**Baseline laboratory test**			
HbA1c (NGSP), %	6.20 (5.73–6.83)	5.80 (5.53–6.20)	0.024 *
HbA1c (IFCC), mmol/L	44.00 (39.25–51.25)	40.00 (37.00–44.00)	0.014 *
Chloride, mmol/L	107.08 ± 2.66	105.32 ± 2.16	<0.001 *
Urea, mmol/L	6.70 (5.10–8.50)	5.30 (4.10–5.60)	<0.001 *
Creatinine, µmol/L	96.00 (81.50–112.50)	82.00 (72.00–92.00)	0.003 *
eGFR, mL/min/1.73 m^2^	72.00 (60.50–89.50)	88.00 (76.00–90.00)	0.007 *
Total protein, g/L	71.00 (68.00–73.00)	74.00 (72.00–76.00)	<0.001 *
Albumin, g/L	37.00 (34.50–39.00)	40.00 (39.00–42.00)	<0.001 *
Total cholesterol, mmol/L	3.80 (3.25–4.80)	5.00 (4.00–5.70)	<0.001 *
HDL cholesterol, mmol/L	1.11 (0.98–1.27)	1.40 (1.16–1.65)	<0.001 *
LDL cholesterol, mmol/L	2.10 (1.56–2.83)	2.80 (2.08–3.43)	0.004 *
Non-HDL cholesterol, mmol/L	2.60 (2.10–3.65)	3.50 (2.58–4.00)	0.016 *
TMAO, µM	3.64 (3.00–4.31)	1.22 (0.92–2.36)	<0.001 *
**Echocardiographic parameters**			
Diastolic dysfunction	^a^	^b^	<0.001 *
Grade I, n (%)	12 (51.17)	14 (35.90)	
Grade II, n (%)	2 (8.70)	0 (0.00)	
Grade III, n (%)	9 (39.13)	0 (0.00)	
EF (MOD-sp4), %	31.60 (25.35–35.10)	67.65 (64.75–71.03)	<0.001 *
EF (Teich), %	35.90 (29.60–40.20)	70.60 (67.00–73.60)	<0.001 *
EDV (MOD-sp4), mL	149.00 (127.00–181.25)	76.50 (64.48–93.00)	<0.001 *
EDV (Teich), mL	212.20 (164.45–244.00)	111.6 (103.7–130.6)	<0.001 *
ESV (MOD-sp4), mL	106.00 (85.75–125.50)	25.50 (19.90–31.23)	<0.001 *
ESV (Teich), mL	137.90 (108.50–166.30)	32.90 (30.00–39.90)	<0.001 *
LA diameter, cm	4.40 (4.10–4.95)	3.60 (3.10–3.93)	<0.001 *
LVEDD, cm	8.40 (7.73–9.03)	6.91 (6.64–7.25)	<0.001 *
IVSs, cm	1.31 ± 0.40	1.50 ± 0.17	0.008 *
LVIDd, cm	6.50 (5.80–6.90)	4.90 (4.70–5.20)	<0.001 *
LVIDs, cm	5.30 (4.80–5.75)	2.90 (2.80–3.20)	<0.001 *
LVPWs, cm	1.32 ± 0.32	1.64 ± 0.26	<0.001 *
IVS fractional thickness, %	25.62 ± 17.41	47.69 ± 17.72	<0.001 *
TAPSE	18.50 (13.25–20.00)	21.00 (19.00–24.00)	<0.001 *
RV S’	10.00 (8.00–11.25)	11.30 (10.38–12.38)	<0.001 *

Continuous variables are presented as mean ± SD or medians with interquartile ranges (IQR), depending on the distribution of the data. EF, ejection fraction; eGFR, estimated glomerular filtration rate; EDV, end-diastolic volume; ESV, end-systolic volume; HbA1c, haemoglobin A1c; HDL, high-density lipoprotein; IFCC, International Federation of Clinical Chemistry; IVSs, interventricular septum thickness at end-systole; IVS fractional thickness, interventricular septum fractional thickness; LA diameter, left atrial diameter; LDL, low-density lipoprotein; LVEDD, left ventricular end-diastolic diameter; LVIDd, left ventricular internal dimension at end-diastole; LVIDs, left ventricular internal dimension at end-systole; LVPWs, left ventricular posterior wall at end-systole; MOD-sp4, modified Simpson’s method; NGSP, National Glycohaemoglobin Standardization Program; RV S’, right ventricular systolic velocity; TAPSE, tricuspid annular plane systolic excursion; Teich, Teichholz method; TMAO, trimethylamine N-oxide. * indicates *p* < 0.05. ^a^ Data not available for 17 participants; ^b^ Data not available for 2 participants. Note: Only variables with statistically significant differences (*p* < 0.05) are displayed. The complete list of all analysed parameters, including non-significant associations, is available in Supplementary Table S2.

**Table 3:** Logistic regression analysis of HFrEF predictors.

Variables	Univariate		Multivariate	
	OR (95% CI)	*p* Value	OR (95% CI)	*p* Value
Age	0.97 (0.91–1.02)	0.257	0.96 (0.84–1.09)	0.538
Gender	1.37 (0.43–4.58)	0.592	17.21 (0.85–660.55)	0.086
BMI	1.05 (0.97–1.15)	0.211		
Ethnicity	1.39 (1.01–1.96)	0.048 *		
Systolic BP	1.00 (0.98–1.03)	0.702		
Diastolic BP	0.98 (0.94–1.02)	0.352		
Heart rate, bpm	1.05 (1.01–1.11)	0.020 *		
Smoking	0.57 (0.31–1.01)	0.058		
Number of cigarettes	1.05 (1.00–1.10)	0.067		
Alcohol	2.41 (1.44–4.29)	0.001 *	1.01 (0.32–3.18)	0.980
Frequency of alcohol consumption/week	3.24 (1.47–8.29)	0.008 *		
Physical activity frequency	1.09 (0.74–1.64)	0.655		
Bowel habit	1.35 (0.83–2.24)	0.232		
Sleep hours	0.84 (0.61–1.14)	0.276		
History of CABG	Not converged	0.989		
History of PCI	Not converged	0.990		
History of myocardial infarction	Not converged	0.992		
History of stroke	1.03 (0.29–3.60)	0.963		
Atrial fibrillation	7.75 (0.72–1054.76)	0.099		
Hypertension	2.31 (0.86–6.53)	0.102		
Dyslipidaemia	0.27 (0.08–0.84)	0.023 *		
T2DM	7.23 (2.80–20.17)	<0.001 *		
CKD	1.48 (1.01–2.26)	0.053		
Obesity	1.84 (0.64–5.59)	0.263		
COPD	3.15 (0.16–465.59)	0.454		
Anaemia	5.39 (0.42–752.63)	0.212		
Gout	2.09 (0.65–7.42)	0.226		
Glucose	1.34 (0.95–1.99)	0.110		
HbA1c (NGSP)	1.93 (1.09–3.78)	0.035 *		
HbA1c (IFCC)	1.06 (1.01–1.13)	0.042 *	1.02 (0.92–1.14)	0.673
Sodium	1.12 (0.90–1.42)	0.319		
Potassium	0.41 (0.16–0.92)	0.061		
Chloride	1.32 (1.09–1.63)	0.006 *		
Urea	1.55 (1.20–2.13)	0.003 *		
Creatinine	1.03 (1.01–1.06)	0.007 *		
eGFR	0.95 (0.91–0.98)	0.004 *	0.96 (0.88–1.03)	0.322
Uric acid	1.00 (1.00–1.01)	0.101		
Total protein	0.80 (0.69–0.91)	0.001 *		
Albumin	0.57 (0.43–0.72)	<0.001 *	0.58 (0.36–0.83)	0.008 *
Globulin	1.02 (0.91–1.15)	0.697		
Total bilirubin	0.94 (0.87–1.01)	0.097		
ALP	1.01 (0.99–1.03)	0.313		
ALT	0.98 (0.95–1.01)	0.251		
AST	1.00 (0.96–1.05)	0.845		
GGT	1.01 (0.99–1.02)	0.295		
Urine albumin	1.00 (1.00–1.00)	0.617		
Urine creatinine	1.00 (1.00–1.00)	0.823		
Total cholesterol	0.42 (0.25–0.67)	<0.001 *		
Triglyceride	0.79 (0.40–1.47)	0.468		
HDL cholesterol	0.03 (0.00–0.16)	<0.001 *	1.09 (0.04–17.19)	0.953
LDL cholesterol	0.47 (0.27–0.79)	0.006 *		
Non-HDL cholesterol	0.55 (0.34–0.84)	0.010 *		
TMAO	2.13 (1.48–3.31)	<0.001 *	1.85 (1.20–3.37)	0.016 *
Diastolic dysfunction	5.77 (2.88–15.02)	<0.001 *	5.19 (1.84–22.50)	0.007 *
EF (MOD-sp4)	0.74 (0.55–0.84)	<0.001 *		
EF (Teich)	0.71 (0.40–0.83)	<0.001 *		
EDV (MOD-sp4)	1.12 (1.07–1.21)	<0.001 *		
EDV (Teich)	1.09 (1.05–1.15)	<0.001 *		
ESV (MOD-sp4)	Not converged	0.998		
ESV (Teich)	1.18 (1.09–1.35)	<0.001 *		
Aortic root diameter	0.79 (0.27–2.27)	0.658		
IVSd	1.37 (0.22–9.10)	0.734		
IVSs	0.09 (0.01–0.43)	0.002 *		
LVPWd	1.09 (0.12–9.74)	0.935		
LVPWs	0.01 (0.00–0.09)	<0.001 *		
RVDd	1.27 (0.64–2.56)	0.494		
IVS fractional thickness	0.93 (0.90–0.96)	<0.001 *		
TAPSE	0.79 (0.68–0.89)	<0.001 *		
RV S’	0.61 (0.44–0.79)	<0.001 *		

Values are presented as Odds Ratios (OR) with 95% Confidence Intervals (CI) and corresponding *p* values. ALP, alkaline phosphatase; ALT, alanine transaminase; AST, aspartate aminotransferase; BMI, body mass index; BP, blood pressure; CABG, coronary artery bypass grafting; CKD, chronic kidney disease; COPD, chronic obstructive pulmonary disease; EF, ejection fraction; eGFR, estimated glomerular filtration rate; EDV, end-diastolic volume; ESV, end-systolic volume; GGT, gamma-glutamyl transferase; HbA1c, haemoglobin A1c; HDL, high-density lipoprotein; HFrEF, heart failure with reduced ejection fraction; IFCC, International Federation of Clinical Chemistry; IVSd, interventricular septum thickness at end-diastole; IVSs, interventricular septum thickness at end-systole; IVS fractional thickness, interventricular septum fractional thickness; LA diameter, left atrial diameter; LDL, low-density lipoprotein; LVEDD, left ventricular end-diastolic diameter; LVIDd, left ventricular internal dimension at end-diastole; LVIDs, left ventricular internal dimension at end-systole; LVPWd; left ventricular posterior wall at end-diastole; LVPWs, left ventricular posterior wall at end-systole; MOD-sp4, modified Simpson’s method; NGSP, National Glycohaemoglobin Standardization Program; PCI, percutaneous coronary intervention; RVDd, right ventricular end-diastolic diameter; RV S’, right ventricular systolic velocity; T2DM, Type 2 diabetes mellitus; TAPSE, tricuspid annular plane systolic excursion; Teich, Teichholz method; TMAO, trimethylamine N-oxide.* indicates *p* < 0.05.

**Table 4:** Multinomial logistic regression of clinical and biochemical predictors across EF categories.

Variable	EF = 30–40%		EF = 20–30%		EF < 20%	
	OR (95% CI)	*p* Value	OR (95% CI)	*p* Value	OR (95% CI)	*p* Value
Age	0.91 (0.79–1.05)	0.213	1.04 (0.89–1.20)	0.632	0.83 (0.57–1.21)	0.330
Gender						
Female	Ref	Ref	Ref	Ref	Ref	Ref
Male	26.51 (0.63–1112.96)	0.086	20.86 (0.51–859.20)	0.109	0.28 (0.00–152.45)	0.690
Alcohol						
Current	Ref	Ref	Ref	Ref	Ref	Ref
Former	1.36 (0.04–44.08)	0.862	1.13 (0.05–25.33)	0.940	1.75 (1.72–1.79)	N/A
Never	1.24 (0.07–20.71)	0.881	0.67 (0.05–8.84)	0.759	Not converged	0.143
eGFR	0.95 (0.87–1.03)	0.177	0.98 (0.91–1.07)	0.698	0.98 (0.89–1.09)	0.754
HbA1c	0.97 (0.86–1.10)	0.632	1.06 (0.94–1.18)	0.339	1.09 (0.90–1.32)	0.355
HDL	0.73 (0.02–35.00)	0.871	1.06 (0.04–28.49)	0.971	7.46 (0.00–12,653.80)	0.596
Diastolic dysfunction	5.83 (1.56–21.82)	0.009 *	5.38 (1.46–19.85)	0.011 *	5.27 (0.59–46.85)	0.136
Albumin	0.56 (0.36–0.89)	0.015 *	0.61 (0.39–0.93)	0.022 *	0.61 (0.28–1.33)	0.212
TMAO	1.83 (1.04–3.23)	0.036 *	2.05 (1.18–3.57)	0.010 *	0.84 (0.21–3.35)	0.807

Values are presented as Odds Ratios (OR) with 95% Confidence Intervals (CI) and corresponding *p* values. Multinomial logistic regression was performed with EF > 40% as the reference group. EF, ejection fraction; eGFR, estimated glomerular filtration rate; HbA1c, haemoglobin A1c; HDL, high-density lipoprotein; Ref, reference category; TMAO, trimethylamine N-oxide. * indicates *p* < 0.05; N/A indicates not applicable.

## Data Availability

The original contributions presented in this study are included in the article/supplementary material. Further inquiries can be directed to the corresponding authors.

## Supplementary Materials

The following supporting information can be downloaded at: https://www.mdpi.com/article/10.3390/ijms27020703/s1.
