# Supplementary material for: Gut Microbiota-Derived Metabolite and Heart Failure with Reduced Ejection Fraction (HFrEF): Elevated Trimethylamine N-Oxide (TMAO) as a Potential Biomarker
Source: Int J Mol Sci. 2026 Jan 9;27(2):703. doi: 10.3390/ijms27020703 (PMC12840905; doi:10.3390/ijms27020703)
Supplement: Supplementary file 1 [file ijms-27-00703-s001.zip › ijms-3980200-supplementary.pdf]

Supplementary Table

Supplementary Table S1: Full list of demographics, lifestyle and behaviour, medical history, and medications of patient and control groups.

Characteristics	HFrEF (N=40)	Control (N=41)	*p* value
**Demographics**			
Age, years	60.00 (53.25-64.00)	63.00 (55.00-66.00)	0.334
Male, n (%)	34 (82.93)	33 (80.49)	0.808
BMI, kg/m^2	26.17 (23.54-30.59)	24.49 (23.14-29.05)	0.255
Ethnicity, n (%)			0.070
Malay	22 (55.00)	12 (29.27)	
Chinese	12 (30.00)	20 (48.78)	
Indian	5 (12.50)	9 (21.95)	
Eurasian	1 (2.50)	0 (0.00)	
NYHA class I/II/III, n (%)	25/12/3 (62.50/30.00/7.50)		N/A
Systolic BP, mmHg	129 (117-145)	130 (122-138)	0.977
Diastolic BP, mmHg	75 ± 12	78 ± 11	0.265
Heart rate, bpm	75.50 (68.75-83.50)	70.00 (64.00-78.00)	0.024*
**Lifestyle and Behaviour, n (%)**			
Smoking			0.108
Former	13 (32.50)	8 (19.51)	
Current	10 (25.00)	6 (14.63)	
Never	17 (42.50)	27 (65.86)	
Number of cigarettes, sticks/day	3.50 (0.00-15.50)	0.00 (0.00-5.00)	0.028*
Alcohol			0.002*
Former	4 (10.00)	2 (4.88)	
Current	5 (12.50)	20 (48.78)	
Never	31 (77.50)	19 (46.34)	
Frequency of alcohol consumption/week			0.005*
Rare (<1)	5 (55.56)	20 (90.91)	
Occasional (1-2)	3 (33.33)	1 (4.55)	
Regular (3-6)	0 (0.00)	1 (4.55)	
Daily (7)	1 (11.11)	0 (0.00)	
Physical activity for ≥ 30minutes			<0.001*
< 1 session/week	29 (72.50)	19 (46.34)	
1-3 sessions/week	8 (20.00)	12 (29.27)	
≥ 4 sessions/week	3 (7.50)	10 (24.39)	
Bowel habit			0.691
At least once per day	18 (45.00)	23 (56.10)	
More than once per day	10 (25.00)	10 (24.39)	
Once every 1-2 days	10 (25.00)	7 (17.07)	
Once every ≥ 3 days	2 (5.00)	1 (2.44)	
Sleep hours	6.00 (5.00-7.00)	6.50 (6.00-7.00)	0.170
**Medical history, n (%)**			
History of CABG	9 (22.50)	0 (0.00)	0.004*
History of PCI	17 (42.50)	0 (0.00)	<0.001*
History of myocardial infarction	25 (62.50)	0 (0.00)	<0.001*
History of stroke	6 (15.00)	6 (14.63)	1.000
Atrial fibrillation	3 (7.50)	0 (0.00)	0.050*

Characteristics	HFrEF (N=40)	Control (N=41)	*p* value
Hypertension	32 (80.00)	26 (63.41)	0.159
Dyslipidaemia	28 (70.00)	37 (90.24)	0.045*
T2DM	28 (70.00)	10 (24.39)	<0.001*
CKD	27 (67.50)	18 (43.90)	0.005*
Obesity	11 (27.50)	7 (17.07)	0.389
COPD	1 (2.50)	0 (0.00)	0.990
Anaemia	2 (5.00)	0 (0.00)	0.463
Gout	9 (22.50)	5 (12.20)	0.351
**Medications, n (%)**			
ACEi	16 (40.00)	7 (17.07)	0.041*
ARB	1 (2.50)	5 (12.20)	0.214
ARNI	22 (55.00)	0 (0.00)	<0.001*
β-blocker	35 (87.50)	6 (14.63)	<0.001*
MRA	31 (77.50)	0 (0.00)	<0.001*
Diuretics	26 (65.00)	1 (2.44)	<0.001*
SGLT2 inhibitor	36 (90.00)	1 (2.44)	<0.001*
Biguanide	10 (25.00)	5 (12.20)	0.231
Calcium channel blocker	5 (12.50)	10 (24.39)	0.275
Statins	33 (82.50)	33 (80.49)	1.000
Antiplatelet	24 (60.00)	5 (12.20)	<0.001*
Anticoagulant	4 (10.00)	0 (0.00)	0.118

Continuous variables are presented as mean ± SD or medians with interquartile ranges (IQR), depending on the distribution of the data. ACEi, angiotensin-converting enzyme inhibitor; ARB, angiotensin II receptor blocker; ARNI, angiotensin receptor-neprilysin inhibitor; BMI, body mass index; BP, blood pressure; CABG, coronary artery bypass grafting; CKD, chronic kidney disease; COPD, chronic obstructive pulmonary disease; HFrEF, heart failure with reduced ejection fraction; MRA, mineralocorticoid receptor antagonist; NYHA, New York Heart Association; PCI, percutaneous coronary intervention; SGLT2 inhibitor, sodium-glucose cotransporter 2 inhibitor; T2DM, Type 2 diabetes mellitus. *indicates $P < 0.05$.

Supplementary Table S2: Full list of laboratory and echocardiographic parameters.

Parameters	HFrEF (N=40)	Control (N=41)	*p* value
**Baseline laboratory test**			
Glucose, mmol/L	5.25 (4.90-6.93)	5.45 (4.98-5.90)	0.905
HbA1c (NGSP), %	6.20 (5.73-6.83)	5.80 (5.53-6.20)	0.024*
HbA1c (IFCC), mmol/L	44.00 (39.25-51.25)	40.00 (37.00-44.00)	0.014*
Sodium, mmol/L	140.00 (138.00-140.00)	139.00 (138.00-140.00)	0.390
Potassium, mmol/L	4.40 (3.95-4.60)	4.40 (4.20-4.90)	0.113
Chloride, mmol/L	107.08 ± 2.66	105.32 ± 2.16	<0.001*
Urea, mmol/L	6.70 (5.10-8.50)	5.30 (4.10-5.60)	<0.001*
Creatinine, μmol/L	96.00 (81.50-112.50)	82.00 (72.00-92.00)	0.003*
eGFR, mL/min/1.73m^2	72.00 (60.50-89.50)	88.00 (76.00-90.00)	0.007*
Uric acid, μmol/L	412.00 (340.75-496.00)	379.00 (311.00-435.00)	0.173
Total protein, g/L	71.00 (68.00-73.00)	74.00 (72.00-76.00)	<0.001*

Parameters	HFrEF (N=40)	Control (N=41)	*p* value
Albumin, g/L	37.00 (34.50-39.00)	40.00 (39.00-42.00)	<0.001*
Globulin, g/L	34.00 (31.50-35.50)	32.00 (32.00-36.00)	0.450
Total bilirubin, μmol/L	13.00 (9.00-17.50)	16.00 (11.00-19.00)	0.100
ALP, IU/L	78.00 (66.50-96.00)	78.00 (67.00-91.00)	0.686
ALT, IU/L	19.50 (14.25-34.50)	26.00 (20.00-34.00)	0.104
AST, IU/L	20.00 (16.50-33.50)	24.00 (19.00-31.00)	0.296
GGT, IU/L	28.00 (20.00-63.00)	23.50 (20.00-49.25)	0.486
Urine albumin, mg/L	18.50 (9.00-34.25)	11.00 (7.00-19.00)	0.447
Urine creatinine, μmol/L	9309.00 (5342.00-11639.50)	10330.00 (5904.75-13407.25)	0.540
Total cholesterol, mmol/L	3.80 (3.25-4.80)	5.00 (4.00-5.70)	<0.001*
Triglyceride, mmol/L	1.10 (0.80-1.65)	1.30 (0.90-1.80)	0.629
HDL cholesterol, mmol/L	1.11 (0.98-1.27)	1.40 (1.16-1.65)	<0.001*
LDL cholesterol, mmol/L	2.10 (1.56-2.83)	2.80 (2.08-3.43)	0.004*
Non-HDL cholesterol, mmol/L	2.60 (2.10-3.65)	3.50 (2.58-4.00)	0.016*
TMAO, μM	3.64 (3.00-4.31)	1.22 (0.92-2.36)	<0.001*
**Echocardiographic parameters**			
Diastolic dysfunction	[a]	[b]	<0.001*
Grade I, n (%)	12 (51.17)	14 (35.90)	
Grade II, n (%)	2 (8.70)	0 (0.00)	
Grade III, n (%)	9 (39.13)	0 (0.00)	
EF (MOD-sp4), %	31.60 (25.35-35.10)	67.65 (64.75-71.03)	<0.001*
EF (Teich), %	35.90 (29.60-40.20)	70.60 (67.00-73.60)	<0.001*
EDV (MOD-sp4), ml	149.00 (127.00-181.25)	76.50 (64.48-93.00)	<0.001*
EDV (Teich), ml	212.20 (164.45-244.00)	111.6 (103.7-130.6)	<0.001*
ESV (MOD-sp4), ml	106.00 (85.75-125.50)	25.50 (19.90-31.23)	<0.001*
ESV (Teich), ml	137.90 (108.50-166.30)	32.90 (30.00-39.90)	<0.001*
Aortic root diameter, cm	3.08 ± 0.46	3.11 ± 0.37	0.809
LA diameter, cm	4.40 (4.10-4.95)	3.60 (3.10-3.93)	<0.001*
LVEDD, cm	8.40 (7.73-9.03)	6.91 (6.64-7.25)	<0.001*
IVSd, cm	0.99 (0.82-1.30)	1.03 (0.93-1.10)	0.756
IVSs, cm	1.31 ± 0.40	1.50 ± 0.17	0.008*
LVIDd, cm	6.50 (5.80-6.90)	4.90 (4.70-5.20)	<0.001*
LVIDs, cm	5.30 (4.80-5.75)	2.90 (2.80-3.20)	<0.001*
LVPWd, cm	0.84 (0.76-1.10)	0.95 (0.86-1.03)	0.274
LVPWs, cm	1.32 ± 0.32	1.64 ± 0.26	<0.001*
RVDd, cm	2.14 ± 0.65	1.85 ± 0.44	0.053
IVS fractional thickness, %	25.62 ± 17.41	47.69 ± 17.72	<0.001*
RA area, cm^2	22.00 (19.20-28.00)	N/A	N/A
TAPSE	18.50 (13.25-20.00)	21.00 (19.00-24.00)	<0.001*
RV S’	10.00 (8.00-11.25)	11.30 (10.38-12.38)	<0.001*

Continuous variables are presented as mean ± SD or medians with interquartile ranges (IQR), depending on the distribution of the data. ALP, alkaline phosphatase; ALT, alanine transaminase; AST, aspartate aminotransferase; EF, ejection fraction; eGFR, estimated glomerular filtration rate; EDV, end-diastolic volume; ESV, end-systolic volume; GGT, gamma-glutamyl transferase; HbA1c, haemoglobin A1c; HDL, high-density lipoprotein; IFCC, International Federation of Clinical Chemistry; IVSd,

interventricular septum thickness at end-diastole; IVSs, interventricular septum thickness at end-systole; IVS fractional thickness, interventricular septum fractional thickness; LA diameter, left atrial diameter; LDL, low-density lipoprotein; LVEDD, left ventricular end-diastolic diameter; LVIDd, left ventricular internal dimension at end-diastole; LVIDs, left ventricular internal dimension at end-systole; ; LVPWd; left ventricular posterior wall at end-diastole; LVPWs, left ventricular posterior wall at end-systole; MOD-sp4, modified Simpson's method; NGSP, National Glycohaemoglobin Standardization Program; RA area, right atrium area; RVDd, right ventricular end-diastolic diameter; RV S′, right ventricular systolic velocity; TAPSE, tricuspid annular plane systolic excursion; Teich, Teichholz method; TMAO, trimethylamine N-oxide. *indicates $P < 0.05$; N/A indicates not applicable. [a]Data not available for 17 participants; [b]Data not available for 2 participants.

Supplementary Table S3: Spearman correlation analysis of TMAO and clinical parameters.

Parameters	TMAO	
	**Coefficient (r_s)**	***p* value**
Diastolic dysfunction	0.417	<0.001
EF (MOD-sp4)	-0.580	<0.001
EF (Teich)	-0.557	<0.001
EDV (MOD-sp4)	0.514	<0.001
EDV (Teich)	0.463	<0.001
ESV (MOD-sp4)	0.535	<0.001
ESV (Teich)	0.555	<0.001
LVEDD	0.489	<0.001
LVIDd	0.514	<0.001
LVIDs	0.529	<0.001
IVS fractional thickness	-0.450	<0.001
Albumin	-0.431	<0.001
Age	0.003	0.981
BMI	0.060	0.592
Heart rate	0.165	0.142
Systolic BP	-0.133	0.235
Diastolic BP	-0.091	0.417
Number of cigarettes	-0.078	0.490
Sleep hours	-0.112	0.319
Glucose	0.070	0.536
HbA1c (NGSP)	0.173	0.122
HbA1c (IFCC)	0.217	0.052
Sodium	0.058	0.605
Potassium	-0.282	0.011
Chloride	0.215	0.054
Urea	0.341	0.002
Creatinine	0.089	0.428
Uric acid	0.149	0.183
Total protein	-0.316	0.004
Globulin	0.051	0.649
Total bilirubin	-0.100	0.375
ALP	-0.062	0.581

ALT	-0.225	0.044
AST	-0.198	0.077
GGT	0.008	0.945
Urine albumin	-0.069	0.541
Urine creatinine	-0.125	0.268
Total cholesterol	-0.240	0.031
Triglyceride	-0.094	0.404
HDL cholesterol	-0.231	0.038
LDL cholesterol	-0.237	0.033
Non-HDL cholesterol	-0.223	0.045
eGFR	-0.190	0.089
CKD	0.156	0.164
Aortic root diameter	-0.231	0.038
IVSd	0.133	0.238
IVSs	-0.169	0.131
LVPWd	0.018	0.874
LVPWs	-0.246	0.027
RVDd	-0.122	0.279
TAPSE	-0.216	0.052
RV S′	-0.125	0.264

Supplementary Table S4: Post-hoc analysis of TMAO levels across controls and NYHA functional classes.

Comparison	Z	*p* unadjusted	*p* adjusted
Control vs. NYHA class I	-4.6069	<0.001	<0.001
Control vs. NYHA class II	-4.2518	<0.001	<0.001
Control vs. NYHA class III	-1.4774	0.140	0.837
NYHA class I vs. NYHA class II	-0.6449	0.519	1.000
NYHA class I vs. NYHA class III	0.4670	0.641	1.000
NYHA class II vs. NYHA class III	0.7929	0.428	1.000

Supplementary Table S5: Parameters grouped by domain for Mantel test.

**Domain**	**Parameters**
Demographics	Age, gender, ethnicity, BMI, heart rate, systolic BP, diastolic BP
Clinical parameters	Glucose, HbA1c (%), sodium, potassium, chloride, urea, creatinine, uric acid, total protein, albumin, globulin, total bilirubin, ALP, ALT, AST, GGT, urine albumin, urine creatinine, total cholesterol, triglyceride, HDL cholesterol, LDL cholesterol, non-HDL cholesterol, eGFR, TMAO
Behavioural	Smoking status, number of cigarettes, alcohol consumption, frequency of alcohol consumption, physical activity frequency, bowel habit, sleep hours
Medical history	History of CABG, PCI, myocardial infarction

Medications	ACEi, ARB, ARNI, β-blocker, MRA, diuretics, SGLT2 inhibitor, biguanide, calcium channel blocker, statins, antiplatelet, anticoagulant
Comorbidities	Stroke, atrial fibrillation, hypertension, dyslipidaemia, T2DM, CKD, obesity, COPD, anaemia, gout
